# Etiologies and prognostic factors of leukocytoclastic vasculitis with skin involvement: A retrospective study in 112 patients

**DOI:** 10.1097/MD.0000000000004238

**Published:** 2016-07-18

**Authors:** Kévin Bouiller, Sylvain Audia, Hervé Devilliers, Evelyne Collet, Marie Hélène Aubriot, Vanessa Leguy-Seguin, Sabine Berthier, Philippe Bonniaud, Pascal Chavanet, Jean-François Besancenot, Pierre Vabres, Laurent Martin, Maxime Samson, Bernard Bonnotte

**Affiliations:** a Service de médecine interne et immunologie clinique; b Service de médecine interne et maladies systémiques; c Service de dermatologie; d Laboratoire d’anatomopathologie; e Service de pneumologie; f Service de maladies infectieuses, CHU François Mitterrand, Dijon, France.

**Keywords:** leukocytoclastic vasculitis, predictive factors, prognosis, relapses, single organ vasculitis

## Abstract

In this study, outcomes of patients with leukocytoclastic vasculitis (LCV) were analyzed focusing on clinical, histopathology and laboratory findings, relapses, and survival.

Data from patients with cutaneous vasculitis diagnosed between January 1, 2000, and December 31, 2010, at Dijon University Hospital (France) were retrospectively reviewed. LCV was defined as perivascular neutrophilic infiltrate, endothelial cell nuclear swelling, extravasation of red blood cells, and/or fibrin deposition in vessels. Patients were classified according to the 2012 Chapel Hill Consensus Conference. Relapses were defined as the recurrence of vasculitis symptoms after a period of remission >1 month. Time to relapse and/or death was calculated from the date of diagnosis. Univariate and multivariate (Cox model) analyses were performed.

A total of 112 patients (57 males and 55 females), with a mean age of 60 ± 19 (18–98) years, were analyzed. Overall follow-up was 61 ± 38 months. At diagnosis, all patients had skin lesions, purpura being the most common (n = 83). Lesions were associated with systemic involvement in 55 (51%) patients. Only 41 (36.6%) patients received specific treatment: glucocorticoids in 29 of 41 (70.7%) and immunosuppressants in 9 of 41 (22%). Sixty-two patients (55%) had LCV due to underlying causes, 29 (25.9%) had single-organ cutaneous small vessel vasculitis (SoCSVV), and 21 (18.8%) had unclassifiable LCV. Twenty patients of the cohort (18%) experienced relapse, 14 ± 13 (1–40) months after the diagnosis of LCV. None of the 29 patients with SoCSVV relapsed. Independent risk factors for relapse were vascular thrombosis in the biopsy [hazard ratio (HR) = 4.9; *P* = 0.017], peripheral neuropathy (HR = 9.8; *P* = 0.001), hepatitis (HR = 3.1; *P* = 0.004), and positive antineutrophil cytoplasm antibodies (ANCA, HR = 5.9 *P* = 0.005). In contrast, SoCSVV was a protective factor for relapse (HR = 0.12; *P* = 0.043).

The 1-, 3-, and 6-year overall survival rates were 99%, 83%, and 71%, respectively, with no difference between relapsers and nonrelapsers (*P* = 0.960) or between SoCSVV and unclassifiable LCV (*P* = 0.588).

This study demonstrates that global survival for LCV patients is good but relapses remain frequent, especially when the cutaneous biopsy shows vascular thrombosis, or in patients with peripheral neuropathy or hepatitis. Conversely, SoCSVV is a protective factor for relapse.

## 1 Introduction

Leukocytoclastic vasculitis (LCV) is a clinico-pathological entity that can be induced by a variety of causes, including drugs, infections, and connective tissue diseases; LCV, however, can also be idiopathic.^[1–8]^ LCV is histopathologically defined and characterized by neutrophilic inflammation in postcapillary venules. The features include neutrophilic infiltration, leukocytoclasia, fibrinoid necrosis, and erythrocyte extravasation into the vessel wall. The skin is often the only organ involved, but systemic involvement also occurs in some patients.^[7,9]^ Conversely, skin lesions can be the initial signs of systemic vasculitis, and, as LCV lesions are not specific, these lesions can be observed in several vasculitis entities.^[3,10–13]^ When LCV is identified by the skin biopsy, it is not possible to distinguish between vasculitis limited to the skin and actual or future systemic vasculitis that only affects the skin at that time. Furthermore, few studies have reported the risk of relapse in patients suffering from LCV.^[7,9,11]^

The 2 main classifications for vasculitides are the American College of Rheumatology (ACR) classification criteria^[14]^ and the International Chapel Hill Consensus Conference (CHCC) definitions,^[15]^ the latter being the most commonly used. One of the novelties introduced in the 2012 revised CHCC nomenclature was the recognition of a novel entity entitled “single-organ vasculitis” (SOV) for vasculitides affecting arteries or veins of any size in a single organ, with no features suggesting limited expression of systemic vasculitis. Therefore, cutaneous polyarteritis nodosa is an SOV with medium-size vessel involvement. When the condition is confined to the skin and affects small vessels, the term single-organ cutaneous small vessel vasculitis (SoCSVV) is thus used. Data regarding the prognosis of this new vasculitis entity are currently lacking.^[16]^ Loricera et al^[16]^ studied 60 patients with SoCSVV among 766 patients with cutaneous vasculitis. Their follow-up was short (median: 4 months), but they showed that all patients with SoCSVV had a complete recovery and that only 8% experienced relapse.

In the present study, we assessed a cohort of patients suffering from LCV who were followed in a single university hospital in order to describe their characteristics and outcomes and to identify predictive factors of relapse, with a special focus on SoCSVV.

## 2 Methods

### 2.1 Studied population

Data from patients with vasculitis diagnosed by a histological cutaneous biopsy between January 1, 2000, and December 31, 2010, at Dijon University Hospital (France, 1780-bed university hospital) were reviewed through an analysis of the database of our pathology department. The data were then retrospectively collected from the medical reports. Only patients with biopsy-proven cutaneous LCV were included in this study. LCV was defined by specific features in the skin biopsy: perivascular neutrophilic infiltrate, endothelial cell nuclear swelling, extravasated red blood cells, and/or fibrin deposition in the vessels.

Patients were classified into different groups depending on the definitions of the revised International CHCC^[15]^:

LCV due to an underlying cause:
  Small vessel vasculitis: hypocomplementemic urticarial vasculitis, cryoglobulinemic vasculitis, IgA vasculitis, antineutrophil cytoplasm antibodies (ANCA)-associated vasculitis
  Vasculitis associated with systemic disease: lupus, sarcoidosis, rheumatoid arthritis, and others.
  Vasculitis associated with a probable etiology: cancer-associated vasculitis, hepatitis C virus associated cryoglobulinemic vasculitis, hepatitis B virus associated vasculitis.
SoCSVV was defined as SOV confined to the skin, with no involvement of other organs.
Unclassifiable LCV for patients without an underlying disease but with systemic symptoms ruling out the diagnosis of SoCSVV.

### 2.2 Data collection

Collected data included gender, age, characteristics of skin lesion(s), general, rheumatological, lung, renal, gastrointestinal, neurological, and ear nose and throat (ENT) symptoms.

Laboratory tests were also collected: C-reactive protein, erythrocyte sedimentation rate (ESR), fibrinogen, human immunodeficiency virus (HIV), hepatitis B virus (HBV), and hepatitis C virus (HCV) serology status, serum complement fractions, cryoglobulin level, rheumatoid factor (RF), antinuclear antibody (ANA), ANCA and anti-proteinase-3 (PR3) and anti-myeloperoxidase (MPO) antibodies, serum electrophoresis and immunofixation, serum creatinine level, hematuria, and proteinuria. Skin biopsies were analyzed using optical microscopy and direct immunofluorescence for the assessment of immunoglobulins and complement deposits along the basement membrane and within the blood vessels wall.

Treatment modalities and outcomes were also recorded. Relapses were defined as the recurrence of vasculitis symptoms after a period of remission >1 month. Severe relapses corresponded to the recurrence or new appearance of major organ involvement, for example, the following, if attributable to active vasculitis: 30% increase in serum creatinine level or 25% decrease in glomerular filtration rate within 3 months or histologic evidence of focal necrotizing glomerulonephritis; clinical, radiologic, or bronchoscopic evidence of pulmonary hemorrhage (pulmonary infiltrates were not considered a severe manifestation); threatened loss of vision related to retinal vasculitis; new multifocal neurologic lesions or mononeuritis multiplex; acute vasculitis-related limb ischemia or gangrene; gastrointestinal hemorrhage or perforation; and other manifestations included in the 1996 FFS: proteinuria >1 g/d, cardiomyopathy and/or central nervous system involvement.^[17,18]^

### 2.3 Statistical analyses

Continuous variables are expressed by mean ± standard deviation (SD) and categorical variables are expressed by number (percentage). Dichotomous variables were analyzed by Chi-square tests or Fisher exact tests, the latter being performed when ≥1 expected cell count(s) was <5. Survival analyses were performed from the date of diagnosis, using the Kaplan–Meier method and survivals were compared with log-rank tests. Multivariate analysis was performed using a Cox regression model with stepwise selection of variables. Variables included in the model were clinically relevant and/or had a *P* value <0.2 with log-rank tests. Thresholds were *P* = 0.3 for entry and *P* = 0.15 for exit. Statistical analyses were performed using SAS v9.0 (SAS Institute Inc.) software (Version 9.0). All tests were 2-sided, and *P* < 0.05 was considered statistically significant.

### 2.4 Ethics statement

Our study is a human noninterventional study wherein subjects were not assigned to treatment, subjects were assigned to a diagnosis strategy within current practice, epidemiological methods were used to analyze the data, and information used in the study were collected for clinical care. According to the Public Health French Law (art L 1121-1-1, art L 1121-1-2), approval from Institutional Review Board and written consent are not required for human noninterventional studies. For ethical consideration, this study was also approved by the Institutional Review Board and the Ethics Committee of Dijon University Hospital.

## 3 Results

### 3.1 Patient population

**Figure 1:**
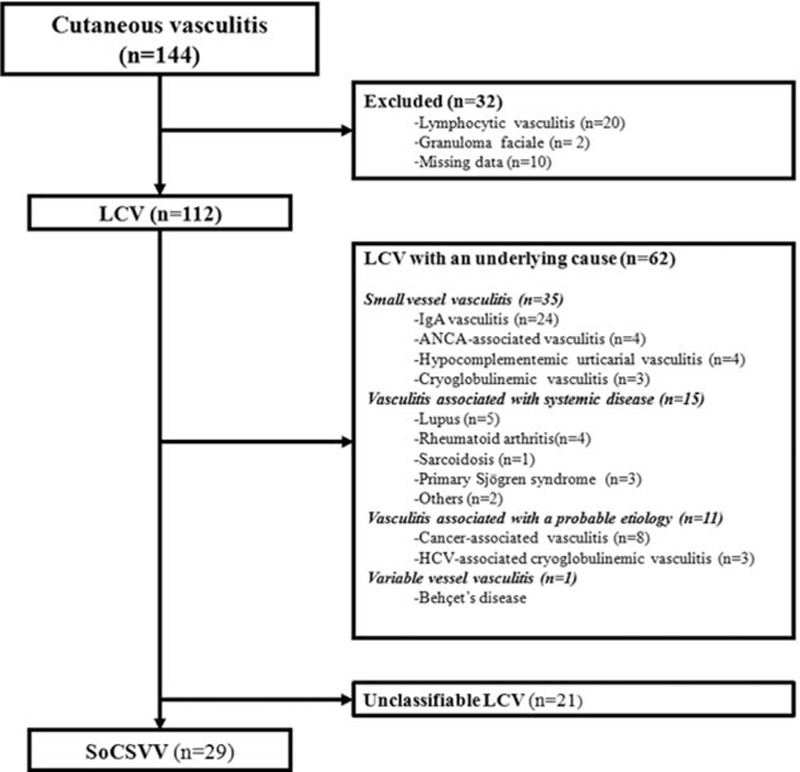
Flow chart of the study. ANCA = antineutrophil cytoplasmic antibody, HCV = hepatitis C virus, LCV = leukocytoclastic vasculitis, SoCSVV = single organ cutaneous small vessel vasculitis.

Between January 1, 2000, and December 31, 2010, a total of 144 patients suffering from cutaneous vasculitis were identified, among whom 32 were excluded from the study: 22 did not meet the inclusion criteria [Granuloma faciale (n = 2) and lymphocytic vasculitis without LCV (n = 20)], and data were lacking for 10 other patients with LCV. Finally, 112 patients were analyzed. Figure 1 shows the flow chart for the study. Notably, 62 (55%) patients had LCV related to an underlying cause: 35 small vessel vasculitis, 15 vasculitis associated with systemic disease, 11 vasculitis associated with probable etiology, and 1 variable vessel vasculitis. Twenty-nine (26%) patients met SoCSVV criteria and 21 (19%) had unclassifiable LCV.

Clinical and biological characteristics of the 112 patients at diagnosis of LCV are summarized in Table 1. Sex ratio (M/F) was 1.04; age at diagnosis was 60.4 ± 18.5 (18–98) years. Cutaneous lesions affected the lower limbs in the majority of patients [79/112 (70.5%)], of whom 45 (57%) had lesions exclusively located on the lower limbs. Purpura was the most frequent type of skin symptom (74.1%). Then, cutaneous necrosis was observed in 22 of 112 (19.6%) patients, pruritus in 10 of 112 (8%), ulcers in 16 of 112 (14.3%), urticaria in 11 of 112 (9.8%), bullae or vesicles in 7 of 112 (6.3%), nodules in 7 of 112 (6.3%), and pustules in 3 of 112 (2.7%).

**Table 1:**
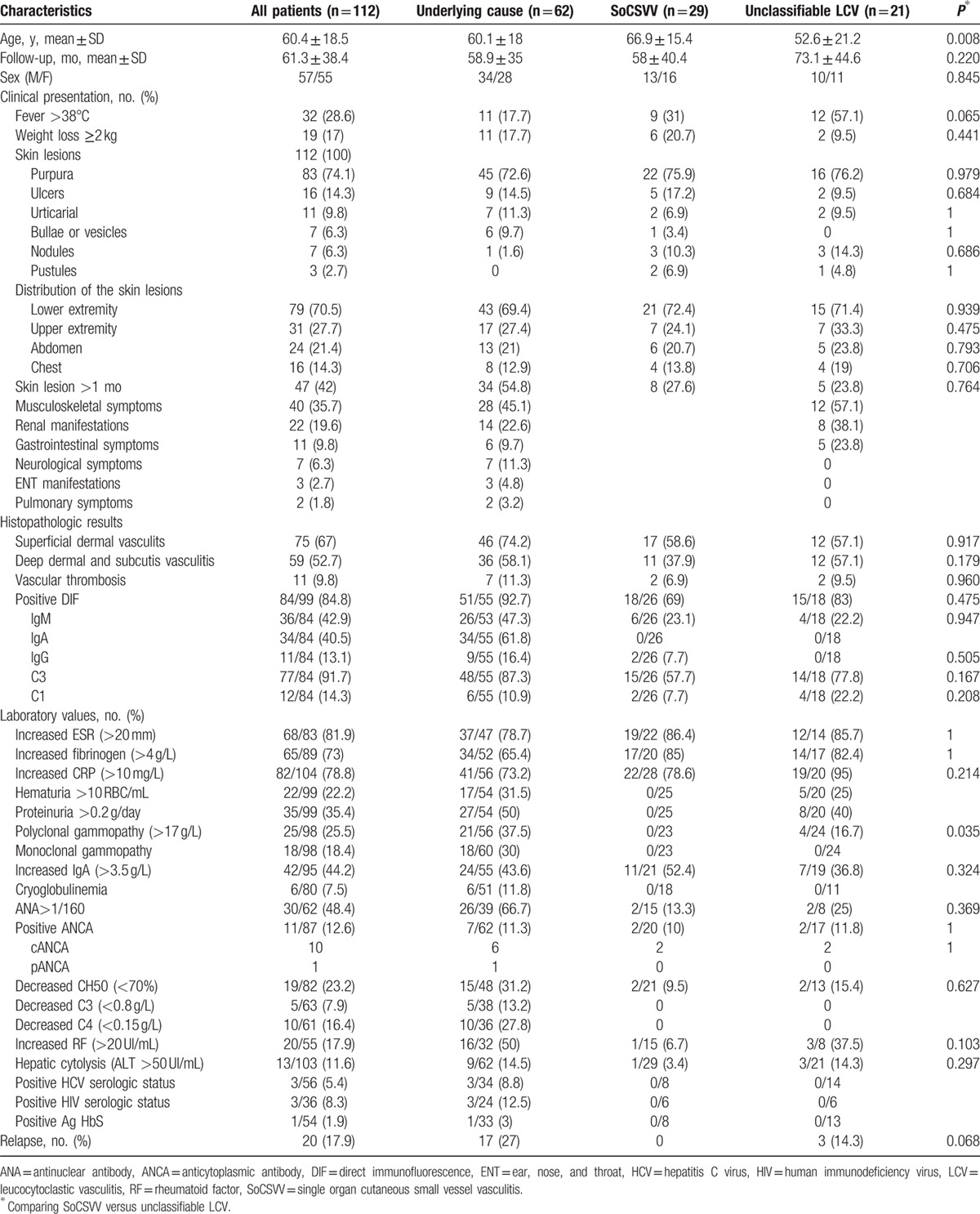
Baseline characteristics of 112 patients with LCV.

**Figure 2:**
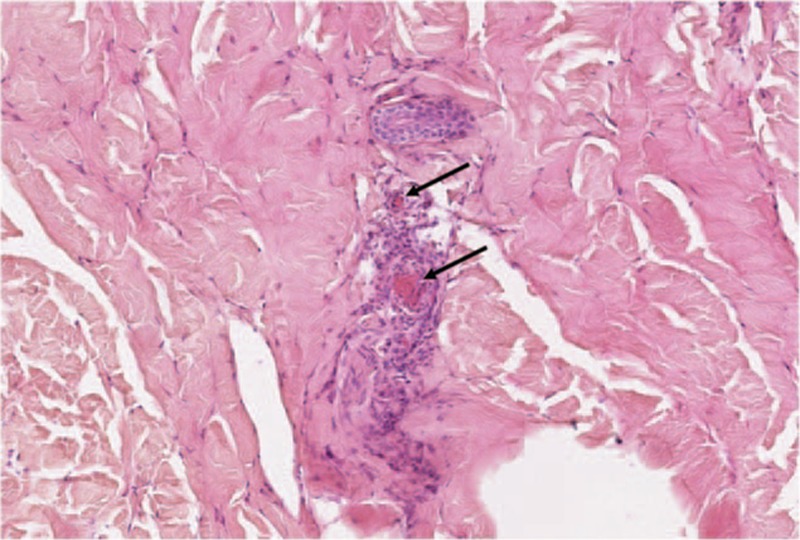
Skin biopsy showing leukocytoclastic vasculitis with thromboses (full arrows).

Histologically, superficial vessels of the skin were affected in 75 patients (67%), whereas deep dermal subcutis lesions were observed in 59 patients (53%) and 27 patients (24%) had vasculitis affecting both superficial and deep dermal vessels. Moreover, infiltration by eosinophils or lymphocytes was observed in 15 patients (13.4%) and 24 patients (21.4%), respectively. Vascular thrombosis (Fig. 2) was observed in 11 patients (9.8%): 7 of 11 patients had purpura, 2 of 11 nodules, 1 of 11 urticaria, and 1 of 11 ulcer, all of these cutaneous lesions occurring in the lower limbs. Five of these 11 patients also had extracutaneous symptoms, mostly arthralgia, and 7 patients had LCV related to an underlying cause: IgA vasculitis (n = 4), granulomatosis with polyangiitis (GPA, n = 1), and solid cancer (n = 2: 1 hepatocellular carcinoma with bone metastases and 1 pulmonary carcinoma).

Direct immunofluorescence was available for 99 of 112 (88.4%) patients and was positive in 84 of 99 (94.4%) skin biopsies. The most common immune protein deposits included C3 (91.7%) and IgM (42.9%). Furthermore, 4 patients (4.8%) had only IgG and 13 patients (15.5%) had only IgM deposits in the skin biopsy.

Extracutaneous involvement was documented in 55 of 112 (49.1%) patients. The organ systems affected during episodes of LCV are listed in Table 1. Notably, the most frequent extracutaneous symptoms were musculoskeletal symptoms (n = 40), renal involvement (n = 22), gastrointestinal symptoms (n = 11), and peripheral neuropathy (n = 7).

A specific treatment was prescribed in 41 patients (36.6%), mainly in patients suffering from LCV related to an underlying disease. Prednisone (70.7%), hydroxychloroquine (14.6%), and colchicine (12.2%) were the most commonly prescribed drugs.

### 3.2 LCV related to an underlying disease

Among the 62 patients with LCV related to an underlying disease, IgA vasculitis was diagnosed in 24 (38.7%) patients, ANCA vasculitis in 4 patients (6.5%) (3 GPA and 1 patient with cANCA vasculitis, not otherwise specified], hypocomplementemic urticarial vasculitis in 4 patients (6.5%), (only 1 patient had anti-C1q antibodies), and cryoglobulinemic vasculitis in 3 patients (4.8%). Vasculitis associated with systemic disease was found in 15 (24.2%) patients: lupus (n = 5), rheumatoid arthritis (n = 4), primary Sjögren syndrome (n = 3), adult-onset Still disease (n = 1), polychondritis (n = 1), and sarcoidosis (n = 1). Eleven patients (17.7%) had vasculitis with a probable etiology: 8 cancer-associated vasculitides (hematological malignancies (n = 4), solid organ neoplasm (n = 4), and 3 patients with hepatitis C virus associated cryoglobulinemic vasculitis. One patient presented Behçet disease.

### 3.3 SoCSVV and unclassifiable LCV

A total of 29 patients (13 men and 16 women) with a mean age of 66.9 ± 15.4 years met the criteria for SoCSVV.^[15]^ These patients were compared with the 21 with unclassifiable LCV. The main results are summarized in Table 1.

Patients with SoCSVV and unclassifiable LCV had mainly purpura lesions (76%), in the lower extremity (72%). Importantly, patients with SoCSVV were older (*P* = 0.008). With regard to potential precipitating factors, a link with the use of drugs (mainly antibiotics and nonsteroidal anti-inflammatory drugs) or acute infection was respectively found in 17.2% and 24.1% of SoCSVV patients and 14.3% and 28.6% of unclassifiable LCV patients (*P* = 0.778 and *P* = 0.724, respectively).

Organ systems affected during episodes of unclassifiable LCV were musculoskeletal symptoms in 12 patients (57.1%), renal manifestations in 8 patients (38.1%), and gastrointestinal symptoms in 5 patients (23.8%). Moreover, 6 patients (28.6%) had only musculoskeletal symptoms.

Inflammatory syndrome was noted in 22 patients (79%) in SoCSVV group versus 19 patients (95%) in unclassifiable LCV. However, patients with SoCSVV had less frequently polyclonal gammopathy (0% vs 17%, *P* = 0.035) than unclassifiable LCV.

Only 4 patients (19%) with unclassifiable LCV and 5 patients (17.2%) with SoCSVV received a specific treatment, mainly prednisone. After a median follow-up of 58 (0–167.5) months, no relapse had occurred in the SoCSVV group, whereas 3 patients (12.5%) experienced a relapse in the unclassifiable LCV group (*P* = 0.068). Importantly, no underlying disease was diagnosed during the follow-up among patients from the SoCSVV and unclassifiable LCV groups.

### 3.4 Outcomes and relapses

After the first LCV flare, remission was obtained for all 112 patients. After a mean follow-up of 61.3 ± 38.4 months (1–167), 20 (17.9%) relapsed 14 ± 13 (1–40) months after the diagnosis: 17 in the underlying cause group (4 IgA vasculitis, 3 HCV infections, 1 nonspecific polyarthritis, 2 GPAs, 1 melanoma with brain metastases, 1 lymphoma, 2 rheumatoid arthritis, 1 essential mixed cryoglobulinemia, 1 polychondritis, 1 Sjögren syndrome), 3 in the unclassifiable LCV group, and none in the SoCSSV group. Characteristics of the first relapse are summarized in Table 2. Notably, 50% of the patients had no treatment or only low doses of prednisone (<10 mg/d) at the time of relapse. Purpura and ulcers were the most prevalent lesions (75%). Systemic involvement was observed in 8 patients (40%). Importantly, severe relapses only occurred in patients suffering from LCV related to an underlying cause (n = 6): mononeuritis multiplex in 2 patients, renal failure (creatinine >140 μmol/L) in 2 patients, pulmonary hemorrhage in 1 patient, and pleural effusion in 1 patient.

**Table 2:**
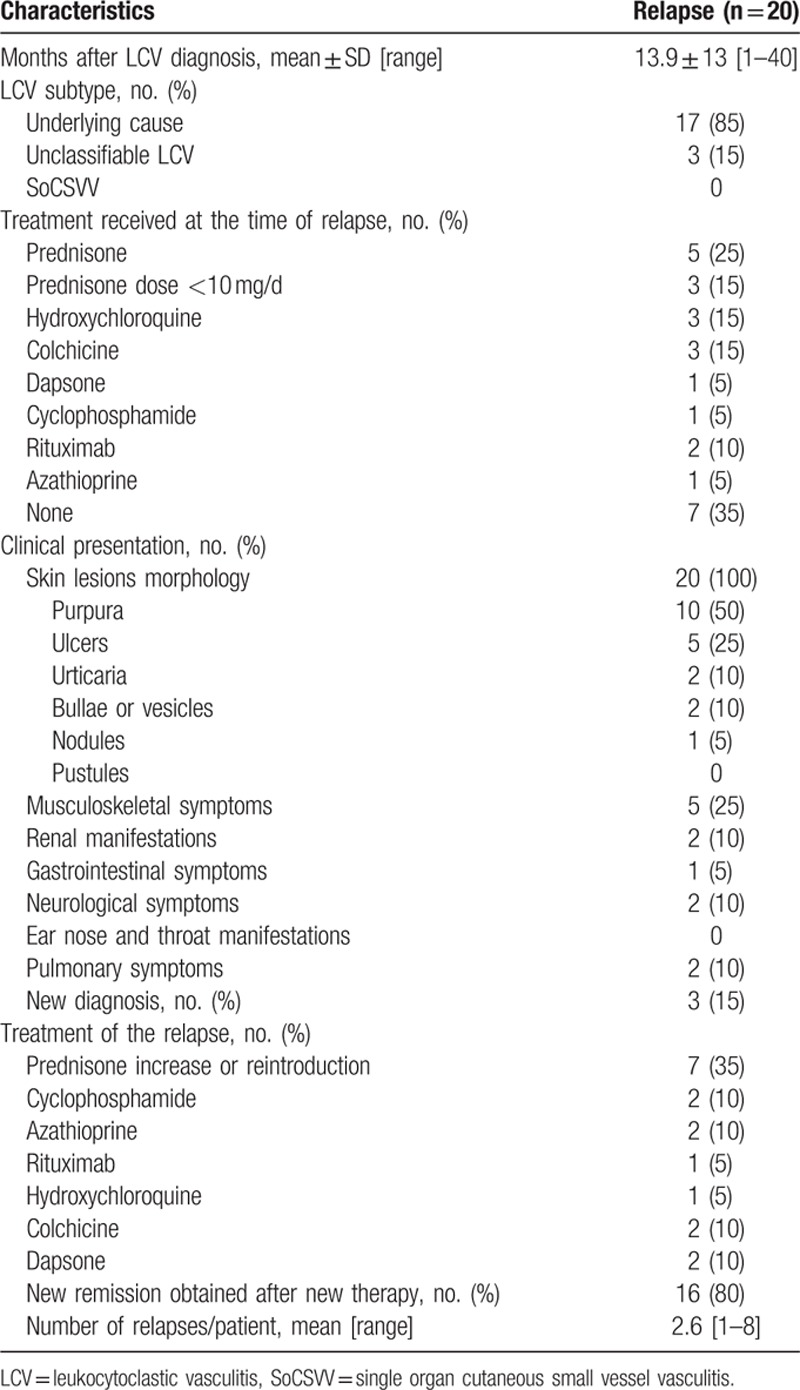
Characteristics of the first relapse of 20 leukocytoclastic vasculitis.

Prednisone was started again or its dose increased to treat 35% of relapses. Immunosuppressants were also prescribed in 5 cases (25%), all in the underlying cause group (Table 2). Thereafter, new remission was obtained in 80% of the patients.

In the univariate analysis, baseline characteristics associated with the occurrence of relapse were neurological symptoms (*P* = 0.017), cutaneous biopsy showing deep dermis lesions (*P* = 0.010) or vascular thrombosis (*P* = 0.020), weight loss >2 kg (*P* = 0.014), positive ANCA detection (*P* = 0.033), increased RF (*P* = 0.001), hepatic cytolysis (*P* = 0.012). In contrast, SoCSVV was a protective factor (*P* = 0.001).

The following variables were analyzed in the Cox regression model: sex (male), deep dermis lesion, vascular thrombosis, weight loss >2 kg, neurological symptoms, positive ANCA, increased RF, hepatic cytolysis, and SoCSVV. Independent risk factors associated with relapse were the presence of vascular thrombosis in the cutaneous biopsy [hazard ratio (HR) = 4.89; 95% confidence interval (95% CI) = 1.32–18.04], hepatic cytolysis (HR = 3.11; 95% CI = 1.03–9.43), positive ANCA (HR = 5.94; 95% CI = 1.70–20.73), neurological symptoms (HR = 9.77; 95% CI = 2.40–39.73), whereas SoCSVV was a protective factor (HR = 0.12; 95% CI = 0.02–0.93) (Table 3).

**Table 3:**
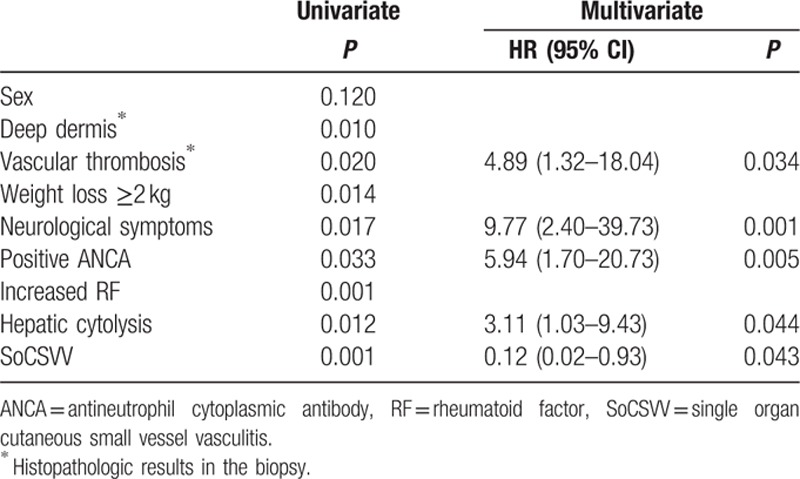
Univariate and multivariate analysis of predictive factors of relapse in patients with leukocytoclastic vasculitis.

### 3.5 Deaths

During the follow-up, 27 (24%) patients died at a mean age of 73.5 ± 16.1 (40.6–101.2) years. Causes of the death were related to systemic vasculitis in 2 patients (7.4%), and not related to vasculitis in the remaining 25 (96.2%) cases. Among the 2 patients who died of active vasculitis, 1 died of cardiac involvement of GPA 15 days after its diagnosis. The other patient died from renal involvement of HCV-associated cryoglobulinemic vasculitis. The 25 other causes of the death were 5 cancers (anal squamous cell carcinoma, B cell lymphoma, lung squamous cell carcinoma, urinary bladder carcinoma, and multiple myeloma), 5 infections [infective endocarditis by methicillin susceptible *Staphylococcus aureus* (n = 2), septic shock caused by *Pseudomonas aeruginosa*, community-acquired pneumonia (n = 2)], 3 cardiovascular events (1 acute coronary syndrome, 1 severe refractory heart failure, 1 acute respiratory failure with chronic obstructive pulmonary disease), 2 renal failures (1 acute renal failure related to hepatorenal syndrome, 1 multifactorial kidney failure), and the cause of death was unknown in 10 patients.

### 3.6 Survival

**Figure 3:**
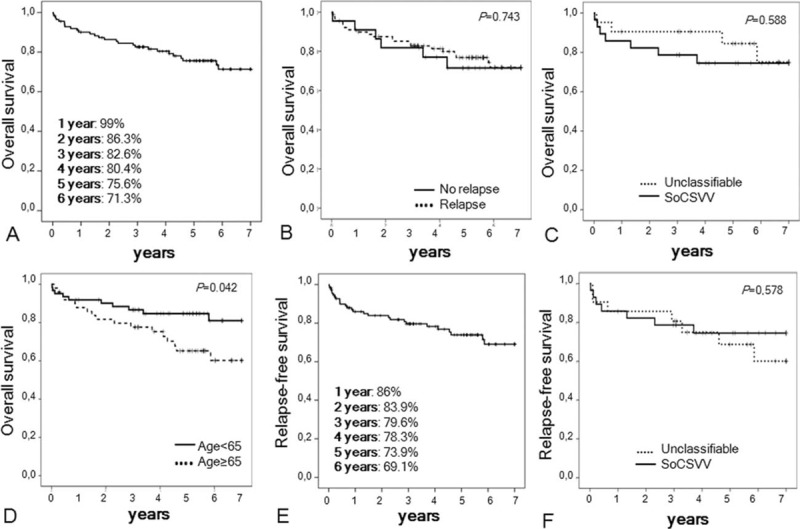
Overall and relapse-free survival analyses of 112 patients with leukocytoclastic vasculitis. *P* is the result of log-rank tests. Data were censored after 7 years of follow-up. SoCSVV = single organ cutaneous small vessel vasculitis.

The 4-, 5-, and 6-year survival rates were 80.4%, 75.6%, and 71.3%, respectively (Fig. 2). Age ≥65 years at baseline was the only factor significantly associated with shorter survival (*P* = 0.042). Notably, survival was not different between the recurrent LCV group and the nonrecurrent LCV group (*P* = 0.743) and between SoCSVV and unclassifiable LCV (*P* = 0.883). Relapse-free survival reached 78.3%, 73.9%, and 69.1% at 4, 5, and 6 years, respectively (Fig. 3).

## 4 Discussion

Herein, we report the long-term follow-up of patients affected by LCV, demonstrating their good overall survival in a large cohort of 112 patients. As already demonstrated in ANCA-associated vasculitis,^[19]^ age ≥65 years at baseline was the only factor significantly associated with shorter survival.

In this study, patients were identified through the analysis of positive skin biopsies. As a result, LCV related to an underlying disease was probably underestimated, as a cutaneous biopsy is not always performed when the diagnosis of vasculitis (e.g., ANCA-associated vasculitis or cryoglobulinemia) has already been made on the basis of other data. Moreover, cutaneous biopsy was not systematically performed in cases of isolated cutaneous lesions that rapidly improved, which could also have resulted in an underestimation of SoCSVV prevalence in our study. These discrepancies, which are mainly related to the retrospective design of our study, did not allow us to accurately compare the frequencies of the different vasculitis entities among LCV. Furthermore, direct immunofluorescence was done in 85% of the biopsies: immunofluorescence was not performed in 3 patients in the SoCSVV group and 3 others in the unclassifiable LCV group, which could have resulted in an underestimation of cases of IgA vasculitis. However, none of the patients classified in the unclassifiable LCV and SoCSVV groups met the criteria of Michel et al^[20]^ for the diagnosis of IgA vasculitis; it is therefore very unlikely that IgA vasculitides were included in the unclassifiable LCV or SoCSVV groups in our study.

In 2012, SoCSVV was individualized by the Chapel Hill Classification Criteria.^[15]^ According to this classification, cutaneous leukocytoclastic angiitis is included under the heading of single-organ vasculitides. This is an important issue, as, due to the restrictive 2012 CHCC definitions, only a few patients with cutaneous LCV would meet the criteria to be included in the SoCSVV category, for which the cause of vasculitis is frequently unknown.^[7,9,16]^

The main clinical feature of LCV in our study, including SoCSVV, was palpable purpura. When comparing SoCSVV with unclassifiable LCV, we found that polyclonal gammopathy was more common in the LCV group, thus leading us to hypothesize that these patients were affected by autoimmune or infectious diseases that had not been diagnosed at the time.^[21]^ Among the 4 patients with hypergammaglobulinemia in the unclassifiable LCV group, no underlying cause was diagnosed during their follow-up. One patient had recently cured infective endocarditis, and another one had alcoholic cirrhosis, thus explaining the occurrence of hypergammaglobulinemia but with no link to the LCV.

Patients with SoCSVV were also significantly older than patients with unclassifiable LCV, and notably, no relapse occurred in the SoCSVV group. In addition, SoCSVV, compared with other LCV, was identified as an independent protective factor of relapse, which highlights the importance of their recent individualization in the 2012 CHCC2.^[15]^ Recently, Loricera et al^[16]^ compared 60 SoCSVV with 250 hypersensitivity vasculitis, as defined by the criteria proposed by Michel et al.^[20]^ They not only showed that patients with SoCSVV were older (*P* = 0.001) but also found that patients with SoCSVV had less anemia (*P* = 0.04) and less leukocytosis (*P* = 0.0003) but a higher percentage of positive rheumatoid factor (P = 0.004) than those with hypersensitivity vasculitis. Even though relapses occurred in 8.3% patients with SoCSVV versus 12.8% patients with hypersensitivity vasculitis, the difference was not statistically significant (*P* = 0.5) in their study.^[16]^ These differences with our study can be explained by a shorter follow-up in the study by Loricera et al (median follow-up of 4 months) than in ours and by the fact that Loricera et al^[16]^ compared SoCSVV with all hypersensitivity vasculitis (with or without extracutaneous symptoms), whereas SoCSVV were compared with unclassifiable LCV in our study.

Systemic involvement was documented in 49.1% of all LCVs in our study, which is in the upper range of previous reports: from 20% to 50% depending on the studies and the recruitment of the patients,^[9,11,13,22]^ arthralgia often being the most frequent extracutaneous symptom.^[11]^ However, for the aforementioned reasons related to the recruitment of our patients and our retrospective design, comparisons with other studies concerning the frequency of extracutaneous symptoms remain hazardous.

In our study, 17.9% of the patients experienced ≥1 relapse. Interestingly, recurrent episodes of LCV were observed in all subtypes of LCV, except SoCSVV. Taking into account the heterogeneity of the definition of relapsing disease and/or the way patients are recruited, our results are consistent with the results of other published studies. In their retrospective analysis of 93 LCV patients, Tai et al^[9]^ reported that 25% had either symptoms lasting ≥3 months or evidence of recurrent symptoms. Ekenstam and Callen^[11]^ showed that 16% of patients with LCV had a relapsing disease, which was defined as 2 or more episodes of LCV lasting less than 3 months for each. Recently, Arora et al^[7]^ also reported in a series of 84 LCV patients who 30% suffered ≥1 episode of LCV.

Predictive factors of relapsing disease in LCV have rarely been studied^[11,13]^ and it is one of the strengths of our study. In the study of Sais et al,^[13]^ which focused on cutaneous vasculitis with a minimal follow-up of 3 years, risk factors associated with chronic disease were cryoglobulins, arthralgia, and normal temperature at diagnosis. Of note, a high incidence of LCV associated with HCV (21.4%) was noticed in this study,^[13]^ thus explaining the association between chronic disease and cryoglobulinemia. In another study focusing on IgA vasculitis,^[23]^ factors associated with relapse were older age, persistent rash, abdominal pain, hematuria, underlying disease at the onset of IgA vasculitis, severity of the leukocytoclasis, and the absence of IgM deposit on the vessel walls.^[23]^ In the study of Alalwani et al,^[24]^ the authors investigated the prognostic value of the type of Ig deposits (IgA, IgG, and IgM) in 218 LCV patients, and demonstrated that IgA (but not IgM and IgG) deposits were associated with a higher risk of renal and gastrointestinal organ involvement. In our study, no correlation was observed between the results of the direct immunofluorescence and the occurrence of LCV relapses, as previously reported.^[10,25]^ In our study, vascular thrombosis in the cutaneous biopsy was associated with relapse. Other risk factors associated with relapse of LCV were hepatic cytolysis, positive ANCA serology, and peripheral neuropathy, all of which are associated with systemic vasculitides related to an underlying disease, thus rationally contrasting with SoCSVV, which was the only protective factor for relapse in our study. Even though relapses have been described in SoCSVV,^[16,26]^ our work shows that the prognosis is better in LCV patients who meet the criteria for SoCSVV. As direct immunofluorescence was done in 85% of biopsies, we performed the analysis of factors associated with relapse after exclusion of the 13 patients for whom immunofluorescence was not available. The results remained similar except for hepatic cytolysis, which tended to be associated with relapse without reaching the level of significance [HR (95% CI): 2.98 (0.95–9.27); *P* = 0.065].

Twenty-seven (24.1%) patients died during our study, which is higher than reported in previous studies (from 2.4% to 25% depending on the studies).^[7,11,27,28]^ This difference can be explained by differences in the duration of the follow-up and in factors worsening the prognosis, such as the age and/or the proportion of patients with underlying systemic vasculitis. In our study, only 2 of 27 deaths were attributable to LCV. Furthermore, none of the 10 patients who died without an identified cause were treated for LCV at the time of death.

LCV is a clinicopathological entity that gathers several vasculitis entities usually classified according to the 2012 revised International CHCC Nomenclature of Vasculitides.^[15]^ However, a few patients cannot be classified in the newly created SoCSVV category, as they have systemic symptoms but do not meet the criteria for other diagnoses. Interestingly, the outcome of these patients seems to be different, with more relapses than in patients with SoCSVV. In any case, overall survival in all types of LCV is excellent, except for patients aged ≥65 years at diagnosis. However, 18% of patients experience relapse(s), especially when vascular thrombosis is identified in the skin biopsy, when ANCA serology is positive, when hepatic liver enzymes are elevated, or when peripheral neuropathy is present at baseline. Among LCV, SoCSVV is a specific entity that is characterized by its good prognosis with a lower risk of relapse. Our results thus demonstrate the importance of the etiological investigations in LCV patients, in order to identify situations associated with severe disease or poorer outcomes, especially relapses.

## Acknowledgment

We thank Philip Bastable for his help in writing the manuscript.
